# Interleukin-10 deficiency impairs regulatory T cell-derived neuropilin-1 functions and promotes Th1 and Th17 immunity

**DOI:** 10.1038/srep24249

**Published:** 2016-04-14

**Authors:** Shimin Wang, Xiang Gao, Guobo Shen, Wei Wang, Jingyu Li, Jingyi Zhao, Yu-Quan Wei, Carl K. Edwards

**Affiliations:** 1 Department of Neurosurgery, State Key Laboratory of Biotherapy/Collaborative Innovation Center for Biotherapy, and West China Hospital, West China Medical School, Sichuan University, Chengdu, 610041, PR China; 2 Institute of Neurosurgery, West China Hospital, West China Medical School, Sichuan University, Chengdu, 610041, PR China

## Abstract

Regulatory T cells (Tregs) expand in peripheral lymphoid organs and can produce immunosuppressive cytokines to support tumor growth. IL-10 abrogation efficiently induces Treg formation but dampens tumoral neuropilin-1 (Nrp-1) Treg signaling, which simultaneously augments Th1 and Th17 immunity. These effects are associated with the plasticity and stability of Tregs and effector T cell functions that can limit tumorigenesis. Within the tumor microenvironment, there appears to be a “mutual antagonism” between immunoenhancement and immunosuppression mechanisms, eventually leading to decreased metastasis. In contrast, tumor progression is paralleled by a reduction in Nrp-1-producing Tregs controlled by the IL-10 and TGF-β1 levels. However, Th1, Th17 and Treg immunity is primarily regulated by IL-10 or Nrp-1 and not TGF-β1 except when combined with IL-10. These results emphasize the important implications for the therapeutic use of Tregs. The number of Treg cells must be maintained in a healthy and dynamic homeostatic range to prevent malignant diseases. Moreover, Treg-mediated immunosuppression can be limited by reducing tumor-derived Treg Nrp-1 levels.

Interleukin-10 (IL-10) is a major anti-inflammatory cytokine that has diverse effects on both innate and adaptive immunity^1^. One report has shown slower tumor growth and enhanced anti-tumor T cell responses in IL-10-KO hosts^2^.

In humans, IL-10 is primarily produced by monocytes and to a lesser extent by type 2 T helper cells (TH2), mastocytes, CD4^+^CD25^+^Foxp3^+^ regulatory T cells, and certain subsets of activated T cells and B cells^3^. IL-10 is a cytokine with multiple pleiotropic effects in immunoregulation and inflammation. IL-10 can inhibit the synthesis of pro-inflammatory cytokines such as IFN-γ, IL-2, IL-3 and TNF-α produced by cells such as Mφ and regulatory T-cells^4^. Moreover, IL-10 can act on regulatory T cells to maintain transcription factor Foxp3 expression and suppressive functions in mice with colitis^5^.

Regulatory T cells (Tregs) are present in tissues throughout the body. They play a crucial role in immunity by preventing autoimmunity and immunopathology and maintaining immunological homeostasis^6^. However, very few studies have examined the plasticity and steady state of Tregs. Tregs present a major barrier to effective anti-tumor immune responses, and to date, their therapeutic use has been impeded by this barrier. Recent studies have shown that human skin has a population of tissue-resident Tregs that produce an elevated level of IL-17 and are functionally defective and phenotypically diverse under inflammatory conditions^7^. Identification of the origination site of Tregs is important because their differentiation into effector lineages modifies their migration, homeostasis and various peripheral functional profiles^8^. The functional properties of different Treg subsets and their immunoregulatory abilities remain elusive. Importantly, the identification of Neuropilin-1 (Nrp-1) on the surfaces of natural and induced Tregs has greatly improved our ability to characterize the two Treg subsets^9^. Foxp3^+^-expressing Tregs isolated from secondary lymphoid organs in C57BL/6 mice contain two subsets: an Nrp-1^hi^ subset (70–80% of total Foxp3^+^ T cells) and an Nrp-1 low subset (20–30% of total Foxp3^+^ T cells) that are identified as natural (nTreg) and induced Tregs (iTreg)^10^. These studies have helped to characterize the specific contributions of these Treg populations because they relate to their differentiation, proliferation, and ability to suppress the immune response^11^. Numerous malignant tumor and endothelial cell phenotypes express various soluble molecules (TGF-β1) that have been shown to interact with these receptors and modulate cancer progression^12^. VEGF_165_ and Semaphorin 3A share overlapping binding domains in the N-terminal region of the b1 domain that compete for binding to Nrp-1 and act in combination with VEGF_165_ to support tumor growth^13^. Nrp-1 is a high-affinity receptor for TGF-β1 on the membrane of tumor cells and can activate the latent form of TGF-β1, which is referred to as the “latency-associated peptide” (LAP)–TGF-β1. This peptide is required to maintain Treg tolerance and to expand their suppressive abilities at inflammatory sites^14^. Although Treg depletion leads to the complete eradication of tumors by maintaining tumor antigens shown to stimulate antitumor immunity, Treg ablation results in the induction of fatal autoimmune disorders^15^. Foxp3 maintenance allows the origin and appropriate numbers of Tregs, and it leads to the preservation of immune homeostasis, specification, and Treg functions; however, Foxp3 does not act alone^16^. Interestingly, there appears to be an Nrp1-dependent augmentation of IL10^+^, ICOS^+^, and CD73^+^ intratumoral Tregs^17^. This population maintains its immune homeostasis and differentiation state via steady-state expression of Foxp3 and its various cofactors into multiple feedback loops programmed by Tregs^9^. Tregs can be recruited by tumor cells to support tumor growth^18^. In addition to Foxp3, Treg suppressive activity correlates with the level of the immunosuppressive cytokine IL-10, which is essential for peripheral tolerance. Furthermore, TGF-β can induce peripheral IL-10-expressing Tregs from Foxp3^+^ and Foxp3- precursor cells to participate in antitumor immune responses by tempering T cell immunity to tumorassociated antigens, thereby dampening successful immunotherapy. TGF-β seems to be essential for the development of IL-10-competent Tregs from CD4^+^ precursor cells regardless of their Foxp3 status^19^.

The present study was designed to analyze the cellular mechanisms of Treg-related tumor immune responses and, in particular, the tumor microenvironment. We investigated Treg immunomodulating cytokines that influenced B16/F10 melanoma tumors in immunocompetent and IL-10 knockout mice. Our data demonstrate and confirm the strong antitumor activities of Tregs and their secreted immunosuppressive cytokines, which constitute an essential counterbalance to adaptive immune responses. The plasticity and stability of Tregs are maintained by restricting the number of Foxp3^+^ Treg cells within a healthy range to prevent malignant diseases.

## Results

### IL-10 Deficiency Inhibits Tumor Growth and Lung Focus Formation *In Vivo*

**Figure 1:**
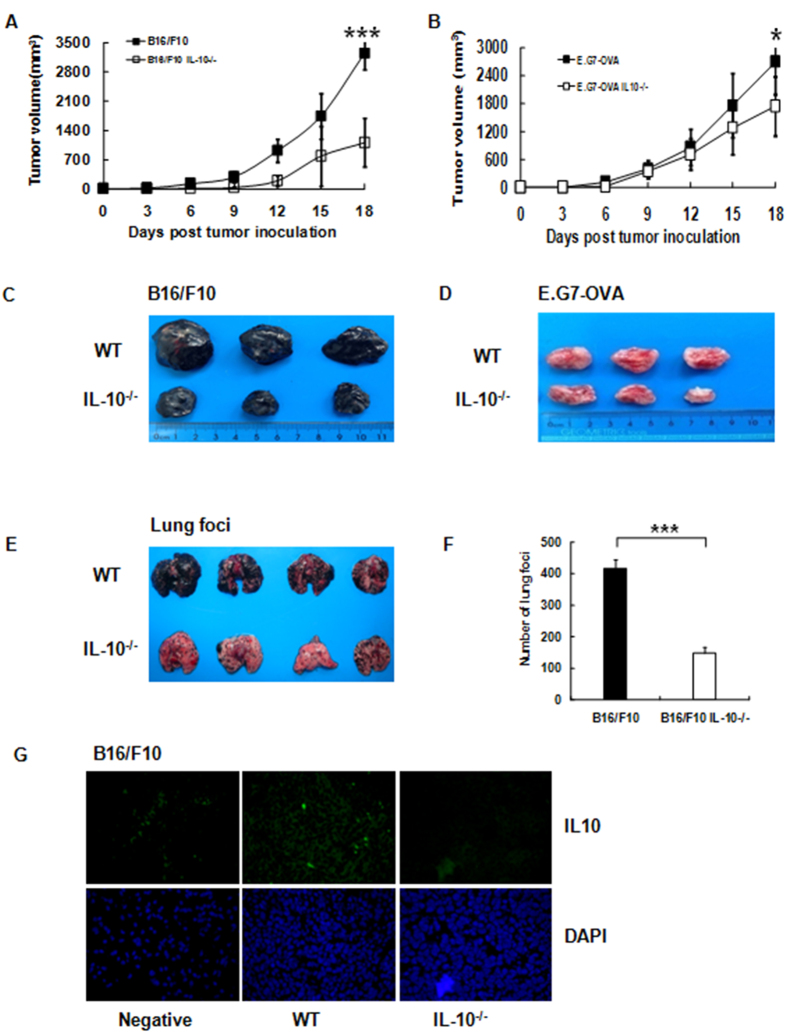
IL-10 deficiency results in decreased tumor growth and focus formation. (**A**,**B**) B16/F10 or E.G7-OVA tumor cells were inoculated s.c. into the left flanks of WT or IL-10^−/−^ C57BL/6 mice. Tumor volumes were monitored and recorded on days +3, +6,+9, +12, +15 and +18 (n = 10–15 mice per experiment, three mice per group at each time point).The mean ± SEM is shown, ***P < 0.001, *P < 0.05, one-way ANOVA. (**C**,**D**) Examples of tumors excised from IL-10^−/−^ and WT mice on day ^+^18; B16/F10 (left) or E.G7-OVA cells (right). (**E**) Melanoma tumor lung foci in WT and IL10^−/−^ mice. B16/F10 cells (3 × 10^5^/100 μl) were injected IV into WT and IL-10^−/−^ mice and counted (d^+^15). (**F**) Bar graph showing representative melanoma tumor lung foci (n = 5–8 mice for IL-10^−/−^ and WT B16/F10 tumor-bearing mice). Error bar(s) represent the mean ± SEM (***P < 0.001), Student’s t-test. (**G**) Immunofluorescent staining of IL-10 on B16/F10 biopsies obtained on d+15 from IL-10^−/−^ and WT tumor-bearing mice. Left, stained with PBS as aa negative control; Middle and Right, stained with anti-IL-10 Mab. Images were obtained using a fluorescence microscope with a 20X objective. Error bar(s) represent the mean ± SEM, n = 3–4 mice. *P < 0.05, **P < 0.01, ***P < 0.001, Student’s t-test or ANOVA.

We took advantage of C57BL/6 IL-10 “gene knockout” mice to conduct a thorough investigation of the roles of IL-10 and Tregs in B16/F10 melanoma. Wild-type (WT) or IL-10 knockout (IL-10^−/−^) mice were implanted with B16/F10 melanoma cells s.c. into the left flank, and tumor volumes were recorded over 15 days. Surprisingly, we observed dramatically decreased (P < 0.001) growth of the melanoma tumor in the IL10^−/−^ mice compared with the WT mice (Fig. 1A). To determine whether these results were restricted to B16/F10 tumors, we performed the same experiment in WT and IL-10^−/−^ mice using the E.G7-OVA lymphoma model (Fig. 1B). Nearly identical results were observed in the IL-10^−/−^ mice, suggesting that complete abrogation of IL-10 led to escape from immune surveillance. The corresponding tumor sizes of the IL10^−/−^ or WT mice are shown (Fig. 1C,D). Next, we addressed the development of lung tumor foci in the IL10^−/−^ or WT mice after B16/F10 injection. The IL-10^−/−^ mice had significantly fewer tumor foci in the lungs compared with the WT mice (Fig. 1E,F). Because B16/F10 cells have been reported to secrete IL-10 under various conditions^20^, we assessed the levels of secreted IL-10 from the IL-10^−/−^ or WT B16/F10 tumors (Fig. 1G). No measurable IL-10 was detected from the IL-10^−/−^ tumor tissue in contrast to the WT tumor-bearing mice.

### Increased IL-10 Production Leads to T Cell Inactivation and Impairment of Adaptive Immunity

**Figure 2:**
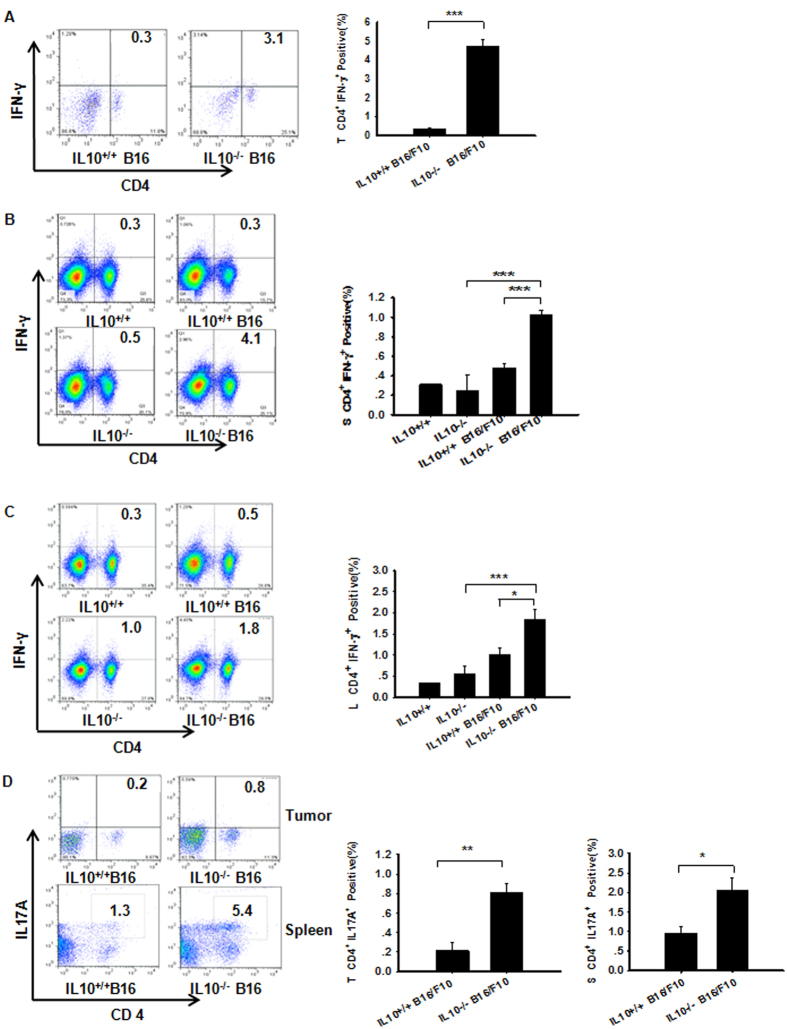
IL-10 deficiency plus B16/F10 implantation results in enchance Th1 and Th17 immunity in melanoma. The percentages of the different T cell subpopulations gated on lymphocytes and defined based on forward and side scatter (FSC/SSC) plots^57^. (**A**–**C**) B16/F10 implantation into IL-10^−/−^ mice resulted in enhanced tumor, spleen and TDLN Th1 cells (CD4^+^IFN-γ^+^) compared with WT B16/F10 mice, as assessed by flow cytometry. (**D**) B16/F10 implantation into IL-10^−/−^ mice resulted in enhanced tumor and splenic Th17 cell (CD4^+^IL-17A^+^) immunity compared with WT B16/F10 mice, as assessed by flow cytometry^58^. Panels A and D: Error bar(s) represent the mean ± SEM, n = 3–4 mice. *P < 0.05, **P < 0.01, ***P < 0.001, Student’s t-test. Panels (**B**,**C**) The mean ± SEM is shown, n = 3–4 mice. *P < 0.05, **P < 0.01, ***P < 0.001, one-way ANOVA.

**Figure 3:**
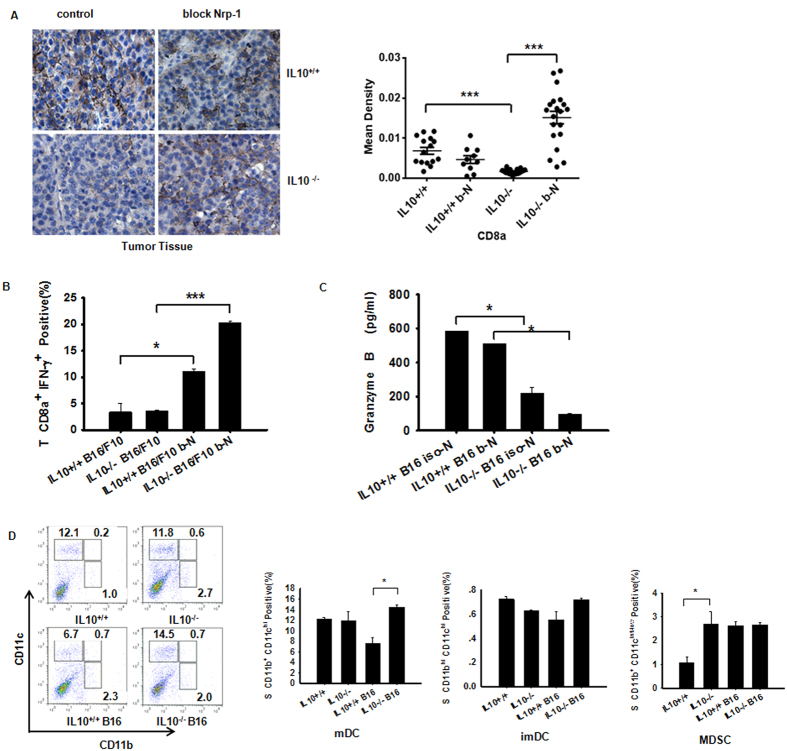
IL-10 interacts with CD8^+^ T cell immunity during tumorigenesis and inhibits DC cell maturation to facilitate tumor growth. (**A**) The blockade of Nrp-1 in melanoma tumor-bearing mice up-regulated CD8a expression in tumor tissues and decreased CD8a protein expression in IL10KO tumor-bearing mice. Representative immunohistochemistry images were obtained using a microscope with a 20X objective. Statistical analysis of the mean IOD for tumor cell proliferation was performed with by Image-Pro Plus software. Nonlinear regressions were performed using GraphPad Prism (San Diego, CA, USA). (**B**) Neutralization of Nrp-1 in IL10^−/−^ or WT mice implanted with B16/F10 tumors augmented CD8a^+^IFN-γ^+^ T cells in the tumor microenvironment compared with the control based on flow cytometry staining. (**C**) Tumor tissues were harvested from tumor-bearing mice on day +15. The tumor-derived cytokine Granzyme B was measured using the mouse multiplexed bead-based immunoassay, MilliplexMapKit (Luminex kit MCD8MAG-48K, Millipore Corporation). (**D**)Mouse spleen-derived DC (spDC) activation and maturation status were detected by flow cytometry. Significantly more mature CD11b^+^CD11c^hi^ cells (mDC, left) were observed in IL10-knockout tumor-bearing mice compared with WT; however, no significant differences were detected in immature CD11b^hi^ CD11c^hi^ (imDC, middle) and CD11b^+^CD11c^int-low^ cells (MDSC, right). Panels (**A**–**D**) The mean ± SEM is shown, n = 3–4 mice. *P < 0.05, **P < 0.01, ***P < 0.001, one-way ANOVA.

Next, we assessed whether IL-10 altered the immune responses in B16/F10 mice, leading to impaired T cell responses. Interestingly, there were significantly increased numbers of CD4^+^IFN-γ^+^ Th1 cells in the tumor, spleen and TDLN harvested from the IL-10^−/−^ B16/F10 mice (Fig. 2A–C). Recent studies have shown that inflammatory Th1 and Th17 responses can facilitate both reduced tumor growth and metastasis^21^. We observed significantly higher tumor and splenic CD4^+^ T cell–derived IL17A production in the IL-10^−/−^ B16/F10 mice (Fig. 2D) but not in the TDLN (data not shown). The neutralization of Nrp-1 in the IL-10^−/−^ or WT B16/F10 mice resulted in elevated tumor-derived CD8a^+^ (Fig. 3A) or IFN-γ protein expression in CD8^+^ T cells (Fig. 3B). In contrast, there was a significant decrease in granzyme B expression (Fig. 3C), which is an important protease that facilitates augmented tumor angiogenesis^22^. These data demonstrated that Nrp-1 inhibited CD8a^+^ protein expression in tumor tissues and limited its function (CD8^+^IFN-γ^+^). The mouse spleen-derived DC (spDC) activation and maturation status were assessed using flow cytometry^23^. Significantly more mature CD11b^+^CD11c^hi^ (mDC) cells were detected in IL-10 knockout tumor-bearing mice compared with WT mice (mDC, Fig. 3D, left). However, no significant differences were detected between immature CD11b^hi^CD11c^hi^ (imDC, Fig. 3D, middle) and CD11b^+^CD11c^int-low^ cells (MDSC, Fig. 3D, right). Mouse mature DCs (mDCs) play a key role in inducing and maintaining antitumor immunity. Additionally, MDSCs are potent inhibitors of T cells in mice, and their inhibition enhances antitumor immunity. Although immature DCs secreted low levels of IL-10, their neutralization was sufficient to promote some DC maturation. Moreover, mature DCs in the presence of an anti-IL-10 mAb were more potent at directing the Th1 differentiation of naive T cells^24^. These data suggest that IL-10 interactions affect CD4^+^ T cell immunity during tumorigenesis and inhibit TH cell immunity to facilitate tumor growth.

### Neuropilin 1 Deficiency in CD4^+^Foxp3^+^ Tregs Impairs B16/F10 Growth through IL-10

**Figure 4:**
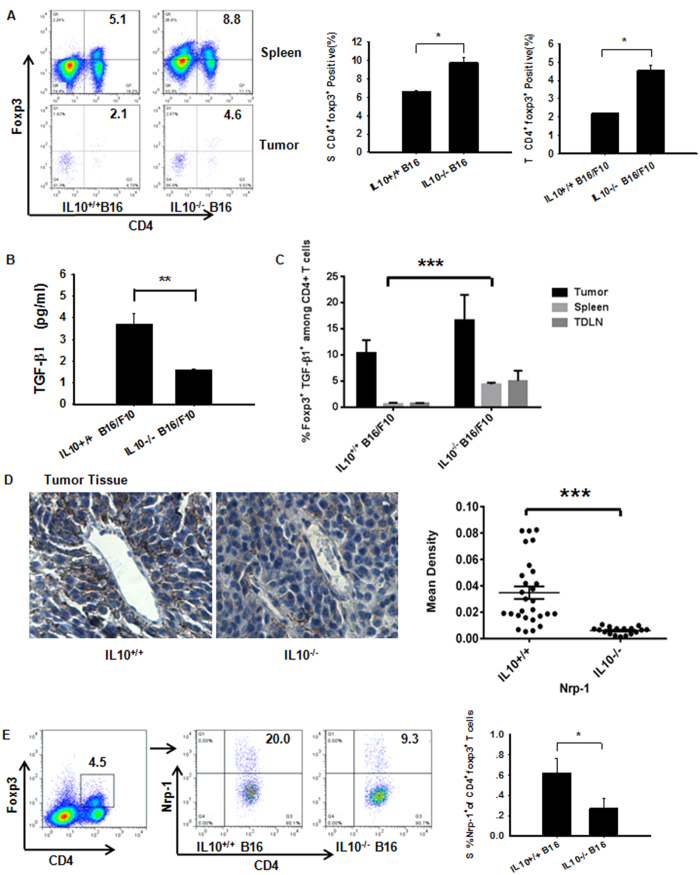
Over-compensation of IL-10 abrogation leads to impairment of treg-Derived Nrp-1 but not TGF-β1 production in B16/F10 tumors. (**A**) Implantation of IL-10-deficient mice with B16/F10 cells resulted in enhanced CD4^+^Foxp3^+^ Tregs in the spleen and tumor on day +15, as assessed by flow cytometry. (**B**) Measurement of TGF-β1 in NP-40-processed B16/F10 tumor tissues (described in the Methods section) obtained from WT and IL-10-deficient tumor-bearing mice. The TGF-β1 cytokine was measured using Luminex (Millipore Merck). (**C**) Among CD4^+^ T cells from IL10^−/−^ tumor-bearing mice, splenic TGF-β1^+^Foxp3^+^ cells were increased compared with WTB16/F10 mice; however, differences in the tumor tissues and TDLNs were not significant. (**D**) IL10 deficiency down-regulated Nrp-1 protein expression in tumor tissues. Representative photomicrographs of B16/F10 tumors harvested on day 15. Left panel, representative immunohistochemistry images obtained using a microscope with a 20X objective. Right panel, statistical analysis of the mean IOD for tumor cell proliferation using Image-Pro Plus software. Nonlinear regression was performed using GraphPad Prism (San Diego, CA, USA). (**E**) Spleen Nrp-1-expressing CD4^+^Foxp3^+^ T cells from IL10^−/−^ tumor-bearing mice were decreased compared with WT B16/F10 mice. Panels (**A,B,D,E**) Evaluated using Student’s t-test to determine statistical significance with *P < 0.05, **P < 0.01, ***P < 0.001between experimental groups. The data are expressed as the mean values ± SEM from n = 3–4 mice. Panel (**C**) Evaluated using ANOVA, ***P < 0.001, ±SEM from n = 3–4 mice.

The presence of Tregs in the periphery is considered crucial for tumor development^3^. However, the mechanism by which Tregs contribute to tumor infiltration through IL-10 production remains elusive. We separately assessed Treg subsets and their abilities to secrete Nrp-1 and TGF-β1. Splenic CD4^+^Foxp3^+^ T cells and tumor CD4^+^Foxp3^+^ T cells obtained from IL-10^−/−^ B16/F10 mice were significantly increased compared with WT mice (Fig. 4A). In contrast, no significant changes were observed in the numbers of tumor-derived macrophages with the M1 or M2 phenotype (data not shown). TGF-β can induce Foxp3 and suppressive Treg cells from naive T cells, and this process can be inhibited^25^. Here, we showed that IL10 deficiency inhibited the tumor secretion of TGF-β (Fig. 4B). However, the number of spleen-derived TGF-β^+^CD4^+^Foxp3^+^ T cells increased compared with WT, with the exception of the tumor and spleen (Fig. 4C). Furthermore, Nrp-1 acts as a key mediator of Foxp3^+^ Treg cell infiltration into the tumor site, resulting in a dampened anti-tumor immune response. Our data showed a clear decrease in Nrp-1 obviously in IL10-deficient tumor-bearing mice (Fig. 4D), and the number of CD4^+^Foxp3^+^Nrp-1^+^ T cells obtained from the spleen (Fig. 4E) was significantly decreased in IL10^−/−^ B16/F10 mice based on flow cytometry analysis^26^. We also observed a strong trend with the TDLN-derived CD4^+^FoxP3^+^Nrp-1^+^ cells in IL-10^−/−^ B16/F10 mice (data not shown). Surprisingly, the Nrp-1-expressing CD4^+^ T cells obtained from the tumor and TDLN were strongly up-regulated in IL10^−/−^ B16/F10 mice, with the exception of the spleen (Supplementary Fig. S1A). This result may be explained by the wide expression of Nrp-1 in lymphoid and myeloid cells. Collectively, our data indicate that Nrp-1 deficiency in CD4^+^Foxp3^+^ regulatory T cells in combination with increased Th1 and Th17 cell immunity results in decreased tumor growth, although elevated expression of Tregs is detected in IL-10^−/−^ B16/F10 mice.

### Nrp-1 Directly Elevates Th1 and Th17 Anti-B16/F10 Immunity

**Figure 5:**
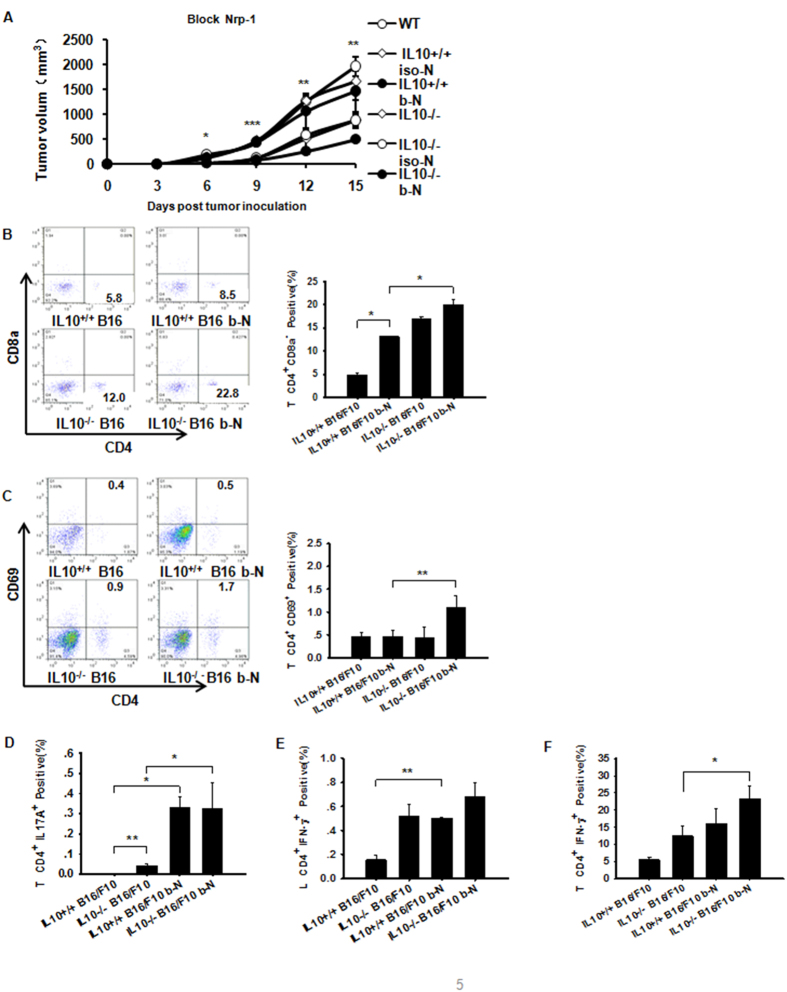
Anti-Nrp-1 reduces melanoma growth in IL-10^−/−^ B16/F10 mice. (**A**) A high-affinity neutralizing anti-Nrp-1 antibody (described in the Methods section) was used to treat WT or IL10^−/−^ B16/F10 tumor-bearing mice. The B16/F10 tumor mass volume was monitored every three days after implantation. (**B**) The majority of B16/F10 tumor-derived CD4^+^CD8a^−^ lymphocytes were increased in the IL10^−/−^ mice after treatment with the anti-Nrp-1 polyclonal Ab, as assessed by flow cytometry. (**C**) Use of anti-Nrp-1 in IL10^−/−^ mice with B16/F10 tumors allowed the augmentation of a CD4^+^CD69^+^-activated T cell population in the tumor microenvironment by flow cytometry. (**D**) Neutralization of Nrp-1 in IL10^−/−^ mice implanted with B16/F10 tumors augmented CD4^+^IL17A^+^ (Th17) T cells in the tumor microenvironment compared with WT mice with B16/F10 tumors alone, as assessed by flow cytometry. (**E**,**F**) Numbers within quadrants represent the percentages of positive cells for a given marker within the lymphocyte gate^59^. Anti-Nrp-1 augmented TDLN or tumor-derived CD4^+^IFN-γ^+^ T cells in WT or IL10^−/−^ B16/F10 mice based on flow cytometry. The bar graph shows data from a representative experiment (mean ± SEM; N = 3–4 mice per group) in which two separate experiments were performed. *P < 0.05, **P < 0.01, ***P < 0.001; assessed by pairwise multiple comparison procedures (Tukey test) and ANOVA.

The results described above suggest that IL-10 deficiency inhibits Nrp-1 expression in B16/F10 tumors. An important question raised by these findings is whether the inhibition of Nrp-1 expression adversely affects T-helper and/or Foxp3^+^ Treg functions. Therefore, we used a neutralizing anti-Nrp-1 Ab^18^ to treat IL10^−/−^ and WT tumor-bearing mice on days +9 and +12 after B16/F10 implantation. On day +15, we harvested the melanoma tumor tissue. Nrp-1 immunohistochemical staining demonstrated that the anti-Nrp-1 antibody clearly decreased Nrp-1 expression in melanoma tumor tissue (Supplementary Fig. S2A). Treatment of the IL-10^−/−^ B16/F10 mice with anti-Nrp-1 significantly reduced the tumor volumes in tumor-bearing mice (Fig. 5A). Anti-Nrp-1 not only allowed the increased expansion of tumor-derived CD4^+^CD8a^−^ T cells(Fig. 5B), but it also activated tumor-derived CD4^+^ T cells (CD4^+^CD69^+^) in IL-10^−/−^ and WT B16/F10 mice (Fig. 5C). Furthermore, blockade of Nrp-1 resulted in significantly augmented tumor-derived CD4^+^IL-17A^+^ (Th17) cells (Fig. 5D). Both TDLN and tumor-derived CD4^+^IFN-γ^+^ (Th1) cells were significantly elevated in IL-10^−/−^ B16/F10 mice treated with the anti-Nrp-1 antibody (Fig. 5E,F). These data demonstrated that Nrp-1 not only decreased the number of activated CD4^+^CD8a^−^ T cells in the tumor *in situ* but was also critically important for Th1 and Th17-mediated anti-tumor immunity.

### Nrp-1 is a Key Mediator of Foxp3+ Treg Infiltration into B16/F10 tumors

**Figure 6:**
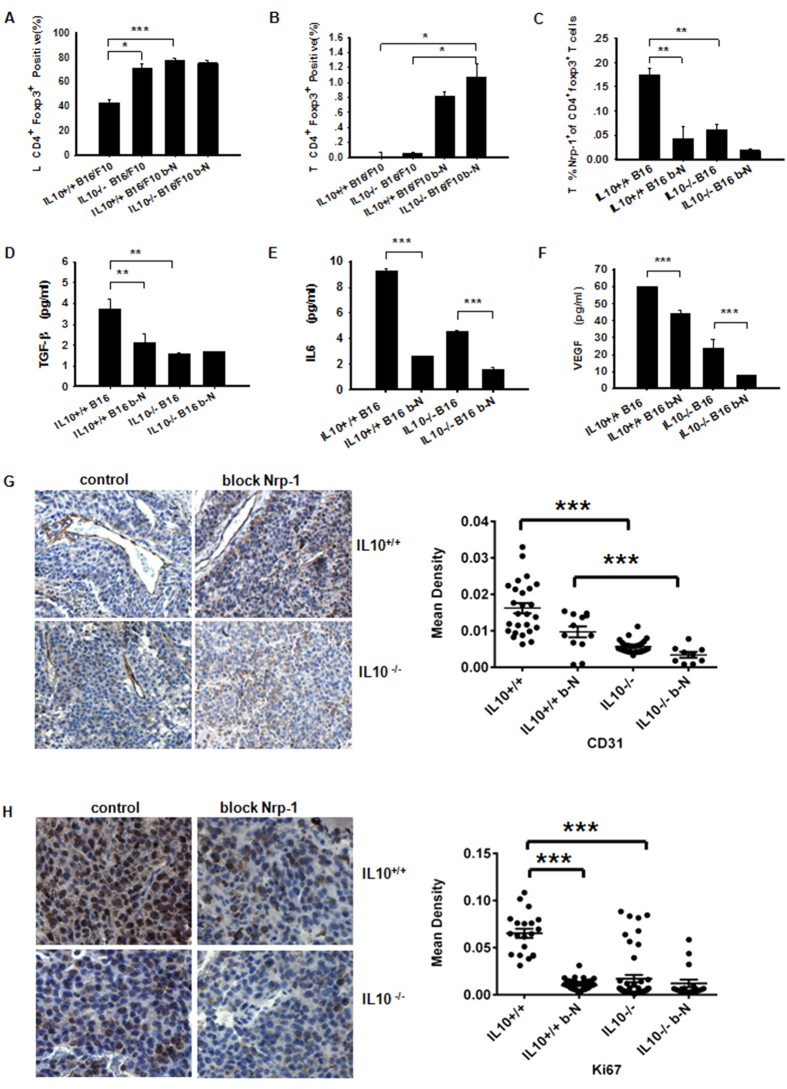
Nrp-1 acts as a key mediator of Foxp3^+^ Treg cell migration to B16/F10 melanomas. (**A,B**) Measurement of B16/F10 TDLN and tumor-derived CD4^+^Foxp3^+^ Treg cells in IL10^−/−^ B16/F10 mice or WT B16/F10 mice after treatment with an anti-Nrp-1 polyclonal Ab by flow cytometry. TDLN and tumor tissue samples were processed as described in the Methods section. (**C**) Measurement of B16/F10 tumor-derived Nrp-1^+^ expression in gated CD4^+^Foxp3^+^ T cells from IL10^−/−^ B16/F10 mice or WT B16/F10 mice after treatment with the anti-Nrp-1 polyclonal Ab by flow cytometry using micro-dissected and digested tumor tissues. (**D–F**) Measurement of TGF-β, IL6 and VEGF expression in NP-40-processed B16/F10 tumor tissues (described in the Methods section) obtained from mice treated with anti-Nrp-1. The cytokines were measured using Luminex (Millipore Merck). (**G**) Blood vessels stained with an anti-CD31 antibody. The images were obtained using a microscope with a 10X objective. (**H**) Compared with the control group, the tumor tissues of mice treated with the anti-Nrp-1 antibody showed much fewer Ki-67-positive cells. Left panel, representative immunohistochemistry images obtained using a microscope with a 20X objective. Right panel, statistical analysis of the mean IOD for blood vessels and tumor cell proliferation performed using Image-Pro Plus software. Nonlinear regressions were performed using GraphPad Prism (San Diego, CA, USA). The data shown are from one representative experiment of two independent experiments with the same results. The results were evaluated using ANOVA for the determination of statistical significance with *P < 0.05, **P < 0.01, ***P < 0.001 between experimental groups. The data are expressed as the mean values ± SEM from n = 4–6 mice.

Next, we assessed tumor-derived Nrp-1-specific Foxp3^+^ Treg cell phenotypes during tumorigenesis. IL-10^−/−^ B16/F10 mice treated with the anti-Nrp-1 antibody exhibited significantly increased numbers of TDLN-CD4^+^Foxp3^+^ Tregs versus IL-10^−/−^ B16/F10 or WT B16/F10 mice (Fig. 6A). We observed almost identical results when measuring tumor-derived CD4^+^Foxp3^+^ T cells (Fig. 6B). Nrp-1-positive tumor-derived CD4^+^Foxp3^+^ (Treg) cells were significantly decreased, as shown by flow cytometry^27^ (Fig. 6C). Tregs utilize TGF-β1 to mediate immunosuppression, and Nrp-1 binds to TGF-β1 to promote Treg activity^28^. Thus, inhibition of either Nrp-1 or IL-10 can affect tumor-derived TGF-β1 levels (Fig. 6D). Treatment of IL-10^−/−^ B16/F10 mice with anti-Nrp-1 significantly reduced tumoral IL-6 levels (Fig. 6E). Because cross-talk relationships between IL-6 and VEGF are important^29^, we measured tumor-derived VEGF and observed reduced VEGF levels in the anti-Nrp-1-treated IL-10^−/−^ B16/F0 mice (Fig. 6F). To investigate whether abrogation of Nrp-1 inhibited tumor growth by suppressing angiogenesis, we used an anti-CD31 antibody to stain melanoma tumor sections. As shown in Fig. 6G, the mean density of tumor blood vessels in the blocked Nrp-1-treated tumors was clearly lower than the mean density in the control group. These results indicated that down-regulation of Nrp-1 inhibited melanoma tumor growth by suppressing tumor angiogenesis. Ki-67 immunohistochemical staining was used to assess the antitumor efficacy on tumor cell proliferation. In this experiment, tumor tissues from the mice treated with the anti-Nrp-1 antibody exhibited fewer Ki-67-positive cells (Fig. 6H). Use of the anti-Nrp-1 antibody resulted in a significantly augmented number of CD4^+^TGF-β1^+^ Th3 cells in the TDLN and tumor tissue (Supplementary Fig. S3A,B) and tumor-derived TGF-β1-producing Treg cells (Supplementary Fig. S3C) from IL10^−/−^ B16/F10 and WT B16/F10 mice. Our data suggested that Nrp-1 expression was more closely related to the Th3 levels and TGF-β1-producing Tregs than IL-10 levels. Collectively, our results indicated that neutralization of Nrp-1 allowed for more impairment of Treg-mediated immunosuppression during tumorigenesis than any other Treg-secreted cytokine.

### TGF-β Signaling is Not Required for the Suppression of Th1 and Th17 Cell-Mediated Inflammation in Contrast to IL-10

**Figure 7:**
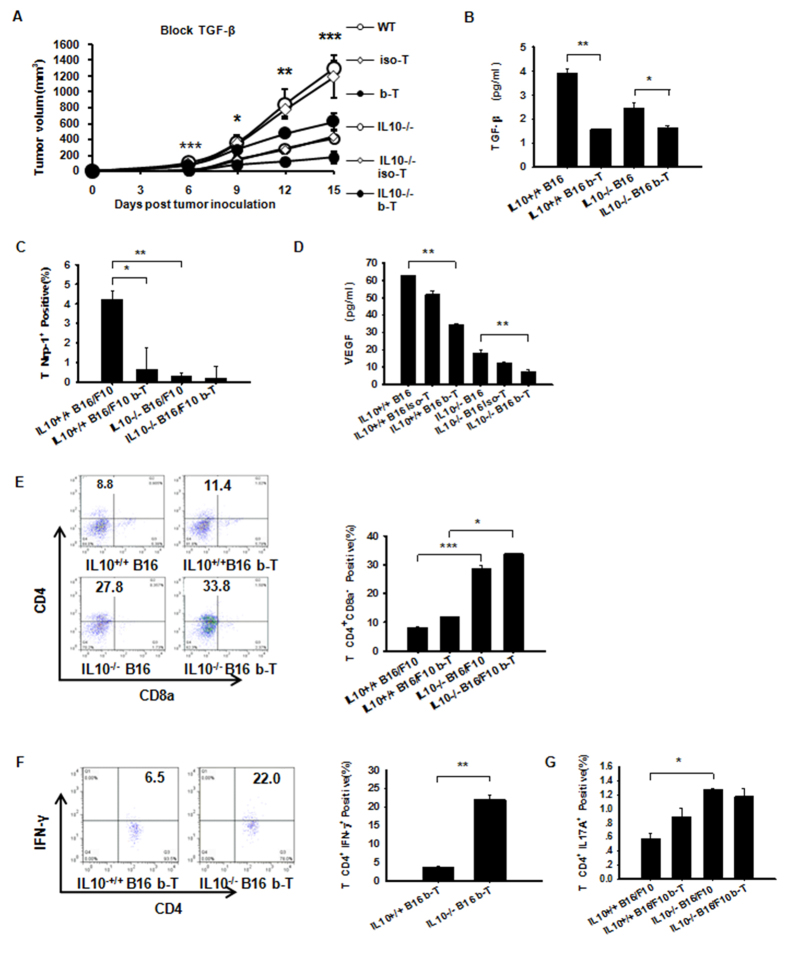
IL-10 but not TGF-β plays the primary role in Th1 and Th17 expansion in B16/F10 tumors and Peripheral Immune Organs. (**A**) A neutralizing anti-TGF-β antibody (described in the Methods section) was used to treat WT or IL10^−/−^ B16/F10 tumor-bearing mice. The tumor mass volume was monitored every three days after implantation. (**B**) TGF-β1 from NP-40-processed B16/F10 tumor tissues was measured using Luminex. Dual inhibition of IL-10 and TGF-β resulted in significantly lower levels of tumor-secreted VEGF protein in B16/F10 tumor-bearing mice. (**C**) Tumor-derived WT B16/F10-derived Nrp-1 levels were elevated compared with IL10^−/−^ B16/F10 mice treated with anti-TGF-β or IL10^−/−^ B16/F10 mice by flow cytometry. (**D**) VEGF from NP-40-processed B16/F10 tumor tissues measured using Luminex. (**E**) The absence of IL-10 or blocked TGF-β antibody had a more pronounced effect on CD4^+^ CD8a^−^ T cells in B16/F10 tumors. (**F**) Anti-TGF-β in IL10^−/−^ B16/F10 mice allowed for the expansion of tumor IFN-γ-expressing CD4^+^ (Th1) populations measured by flow cytometry. (**G**) Anti-TGF-β did not allow the expansion of tumor CD4^+^ IL17A^+^ (Th17) populations in IL10^−/−^ B16/F10 mice measured by flow cytometry. The data shown are from one representative experiment of two independent experiments with same results and were evaluated using ANOVA for the determination of statistical significance with *P < 0.05, **P < 0.01, ***P < 0.001between experimental groups. The data are expressed as the mean values ± SEM from n = 4–6 mice.

Whether the relationship between IL-10 and TGF-β1 together with Treg-derived Nrp-1 involvement can drive local immunosuppression and lead to tumor growth is unknown^14^. Therefore, we treated IL-10^−/−^ or WT tumor-bearing mice intravenously with an anti-TGF-β Ab (described in the Methods section). The tumor volume decreased significantly in IL10^−/−^ B16/F10 mice treated with anti-TGF-β Ab compared with WT mice (Fig. 7A). On day +15, the tumor-secreted TGF-β1 levels in the IL-10^−/−^ and WT B16/F10 mice were both significantly reduced after TGF-β neutralization (Fig. 7B). To address the effects of anti-TGF-β on Nrp-1, Nrp-1 expression was measured by flow cytometry. Our data showed that TGF-β increased intratumoral Nrp-1 expression, thereby supporting tumor growth (Fig. 7C). Conversely, anti-TGF-β significantly decreased tumor VEGF in the IL-10^−/−^ B16/F10 mice (Fig. 7D). Nrp1 is a receptor for VEGF that regulates vascular development and cell motility. Moreover, TGF-β and VEGF_165_ can competitively bind to Nrp-1^27^. After blocking TGF-β, no differences were detected in tumor-derived CD4^+^CD8^−^ T cells compared with untreated cells. These results indicate that IL-10 but not TGF-β plays a role in elevating CD4^+^ CD8^−^ T cells in tumor tissue (Fig. 7E). Similarly, tumor-associated IFN-γ-expressing CD4^+^ (Th1) cells were significantly increased in WT and IL10^−/−^ B16/F10 mice after anti-TGF-β injection (Fig. 7F). However, there was no change in the number of tumor-derived CD4^+^IL17A^+^ (Th17) cells (Fig. 7G). This finding supports a scenario in which TGF-β combined with IL-10 induced Th17 cell production^28^; in turn, Th17 cell-mediated inflammation required IL-10 signaling, especially in regulatory T cells^21^. Our data suggest that IL-10 is closely linked to tumor-associated CD4^+^ populations expressing either IFN-γ or IL-17A, whereas the presence of TGF-β appears to be of secondary importance.

### TGF-β facilitates tumor growth by increasing tumor-derived Nrp-1^+^ Treg cells and plays a less important role in the formation of Treg cells than IL-10

**Figure 8:**
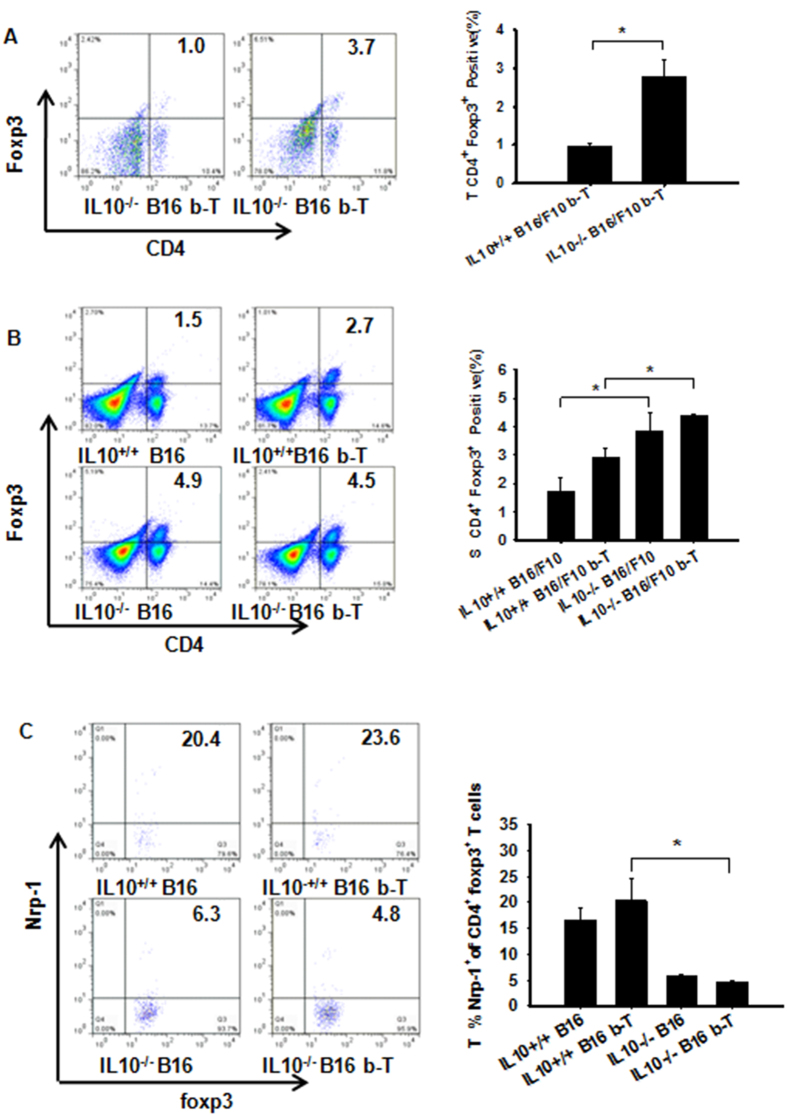
TGF-β-facilitated tumor growth through increased tumor-derived Nrp-1^+^ Treg cells is less important than IL10-affected Treg cell formation. (**A,B**) Percentages of the different T cell subpopulations gated on lymphocytes (FSC/SSC). IL-10 was more important than TGF-β for increasing the CD4^+^Foxp3^+^iTreg cell population in the tumor and spleen microenvironment, as measured by flow cytometry. (**C**) The frequency of Nrp-1–expressing Foxp3^+^ Treg cells from WT or IL10^−/−^ tumor-bearing mice blocked with or without TGF-β1 was determined within tumors or spleens by flow cytometry gating on CD4^+^Foxp3^+^cells^32^. These data show that TGF-β increased the ability of tumor-derived Nrp-1^+^CD4^+^Foxp3^+^T cells to support tumor growth. Panel (**A**) Evaluated using Student’s t-test to determine statistical significance with *P < 0.05 between experimental groups. The data are expressed as the mean values ± SEM from n = 3–4 mice. Panels (**B**,**C**) Evaluated using ANOVA, *P < 0.05, ±SEM from n = 3–4 mice.

The usage of anti-TGF-β allows the stimulation and/or differentiation of Treg populations in the periphery during B16/F10 tumor progression. TGF-β is required for the generation and maintenance of nTregs and iTregs, although this process is not stable^30^. These data raise the question of how the treatment affects tumoral and peripheral Nrp-1 expression. Tumor and spleen-derived CD4^+^Foxp3^+^ Treg cells obtained from anti-TGF-β-treated IL-10^−/−^ B16/F10 mice were significantly increased (Fig. 8A,B). Conversely, Nrp-1-positive tumor-derived Treg cells were decreased (Fig. 8C). Some studies have shown that Nrp-1 has a co-receptor function for TGF-β1 on the membrane of cancer cells and enhances responses to both latent and active TGF-β^14^. Surprisingly, abrogation of IL-10 led to the promotion of tumor-associated Nrp-1 expression on CD4^+^ T cells only after TGF-β Ab treatment (Supplementary Fig. S4A). We also observed that IL-10 was much more important than TGF-β for the up-regulation of tumoral Th3 cells (Supplementary Fig. S4B). Th3 cells produce TGF-β and play a critical role in generating CD4^+^Foxp3^+^ Tregs^31^. Thus, the absence of IL-10 induced Treg promotion. However, TGF-β or IFN-γ-producing TDLN Treg cells were reduced in IL-10^−/−^ or WT tumor-bearing mice after anti-TGF-β treatment (Supplementary Fig. S5A,B). These data suggest that TGF-β abrogation results in delayed tumor growth that is dependent on impaired tumoral Nrp-1^+^ Treg levels. Moreover, TGF-β is less necessary than IL-10 for attenuating tumoral and peripheral organ Foxp3^+^ Tregs levels, possibly due to the ability of IL-10 to promote Foxp3^+^ instability to offset TGF-β responses.

## Discussion

Since its discovery, IL-10 has been recognized as an important pleiotropic immunomodulatory cytokine that affects the innate and adaptive immune systems^32^. IL-10 is produced by many cells, and recent key studies have focused on the regulation of IL-10 production by CD4^+^ T cells^33^. The importance of IL-10 is attributed to its role as a mediator of the suppression of Foxp3^+^ and Foxp3^−^ Tregs^19^. However, the mechanisms underlying the anti-tumor immunosurveillance of IL-10, its regulation of Treg functions during tumor initiation and progression, and its ability to control Treg infiltration into tumors remain unclear^34^.

The key observations in our study confirm and extend the current understanding of how the immune system is regulated during tumorigenesis and emphasize the importance of the immune microenvironments of the tumor, spleen, and TDLN and the roles played by IL-10 and TGF-β in regulating Nrp-1-derived Tregs during this process. First, our data indicate that IL-10 is required for the efficient Th1 and Th17 immune inhibition that impairs Treg recruitment to peripheral organs and B16/F10 melanoma tumors. Indeed, these specialized immune cells appear to “compete” with one another, resulting in the progression of endogenously arising skin tumors. Second, we studied different Treg populations in IL-10-deficient mice and the influence of the absence of IL-10 on decreased tumor volumes and foci. Third and most importantly, our data demonstrate that IL-10 deficiency impairs Treg-derived Nrp-1 functions to promote Th1 and Th17 immunity, suggesting that Nrp-1 is a critical molecule in tumor pathogenesis. Tregs can be induced in the spleen, TDLN, and tumor tissue by increasing myeloid-derived suppressor cells in IL-10-knockout mice bearing MC38 colon carcinoma cells^35^. These findings are consistent with our present results showing that Tregs are strongly augmented in the spleen and TDLN. However, our results differ from those of Tanikawa because strong increases in Tregs in the tumor were not observed^35^. In the absence of IL-10, Th1 and Th17 immunity are strongly up-regulated in IL-10^−/−^ B16/F10 tumor-bearing mice. These results suggest that the interrelationships between Tregs and Th1/Th17 promote and dampen tumor immunity. Finally, our results suggest that B16/F10 tumors may preferentially affect immune responses at regional lymph node sites located near the tumor mass and spleen. To study Treg functions, we analyzed TGF-β1 and Nrp-1-producing Treg subpopulations because these mediators have been shown to be critical for normal host responses to cancer^17^,^26^. TGF-β1 has multiple pleotropic functions, most often with opposing effects that appear to depend on the responding cell types^32^. The interrelationships between TGF-β and Tregs appear to be quite complex because IL-2 and TGF-β have been reported to induce naive T cells to become Foxp3^+^ iTregs^36^, whereas IL-6 abolishes the TGF-β1-induced generation of Foxp3^+^ Tregs^37^. We show that blocking Nrp-1 or IL-10 augments Treg levels, whereas TGF-β is a secondary requirement in comparison to IL-10 to limit Treg numbers. We addressed the influence of TGF-β1 on CD4^+^Foxp3^+^ Treg activation by evaluating the effect of IL-10 responses. IL-10 deficiency in tumor-bearing mice has no inhibitory effect on TGF-β1-derived Tregs obtained from TDLN and tumors, and these effects appear to be opposite in the spleen, where there is an overall increase in TGF-β1-producing Tregs. These data indicate that Treg inhibition is paralleled by the reduction in activated Foxp3^+^Nrp-1-producing Tregs during the early stages of tumor progression and is clearly controlled by IL-10 and TGF-β1. Antigen-stimulated Tregs secrete TGF-β, thereby enabling tumor growth; this growth is dampened by Nrp-1^+^ Tregs concomitant with the presence of IL-10. The impairment of tumor growth after treatment with anti-Nrp-1 confirms our finding that IL-10 plays a central role in this process. Other studies have also confirmed the present findings^26^. In addition to TGF-β, IFN-γ and Nrp-1 are crucial for Treg suppression of immune functions and the effects on APCs and other T cells present in the microenvironment^10^. Th1-like Tregs exhibit reduced suppression *in vitro*, which can be reversed by anti-IFN-γ or abrogation of IL-12^38^. Nrp-1 impairment leads to the loss of Treg suppression and subsequent inhibition of tumor growth. We conclude that Nrp-1 is highly expressed by Tregs that control Foxp3^+^ Treg migration into the tumor, and this process is highly dependent on tumor-derived IL-10. Others have shown that Nrp-1 is involved in T cell activation, is constitutively expressed on CD4^+^CD25^+^ Tregs independent of their activation status and that CD4^+^Nrp-1^hi^ T cells promote Foxp3^+^ mRNA expression^11^. These observations support our present findings that the TDLN and spleen express elevated levels of CD4^+^Nrp-1^+^ cells, resulting in the promotion of CD4^+^CD25^+^Foxp3^+^ or CD4^+^Foxp3^+^Treg expression in IL10^−/−^ tumor-bearing mice. Furthermore, our data suggest that Nrp-1-derived Tregs appear to be quite different from CD4^+^Nrp1^+^ T cells and that these cells most likely play different roles in anti-tumor responses. Although the specific contributions of Treg subsets to tolerance and immune regulation remain elusive, our data suggest that the differential expression of Nrp-1 on nTregs and iTregs can be employed to distinguish these cells *in vivo*^9^,^10^. Nrp-1 is highly expressed by Foxp3^+^ Tregs, which appear to regulate immunological anti-tumor control mechanisms in response to tumor-derived VEGF^39^. We show that tumor tissue can secrete Nrp-1 and VEGF to support its growth, and their production closely parallels the co-expression levels of IL-10 and TGF-β. Inhibition of TGF-β or IL-10 resulted in decreased Nrp-1 expression in tumor tissue and reduced tumor-derived VEGF. These results are consistent with previous findings demonstrating that Sema3A and VEGF_165_ share overlapping binding domains in the N-terminal region of the b1 domain and thus may compete for binding to Nrp-1^40^. Tregs maintain homeostasis in immunity; our data suggest that Foxp3^+^ Tregs obtained from IL-10^−/−^ B16/F10 tumor-bearing mice become unstable after antigen stimulation depriving them of Foxp3^+^ expression.

As observed during the process of TH cell differentiation from naive T cells after antigen exposure, Treg cells show adaptive properties by sensing environmental cues^41^. Too few Treg triggers can lead to fatal autoimmune responses, whereas too many triggers can cause immune suppression^42^. To maintain immune homeostasis, our immune system appears to increase Treg numbers (e.g., in response to foreign antigens). In the absence of IL-10, impaired Treg-derived Nrp-1 suppressive functions and Foxp3 expression may be lost, allowing their transformation into effector CD4^+^ T cells to counteract tumor growth. We demonstrate that Nrp-1 inhibition *in vivo* promotes Th1 and Th17 responses while simultaneously leading to increased Treg levels to dampen immunity. We believe that the presence of different immune cell phenotypes leads to mutual antagonism in the tumor microenvironment, resulting in tumor growth inhibition. Although the production of proinflammatory cytokines by Tregs appears to contradict their immunosuppressive function, our data suggest that this result does not necessarily mean that Treg plasticity is unlimited^43^. Our findings are consistent with previous studies showing that Foxp3^+^ Tregs recruited to Th1 inflammatory sites express T-bet and CXCR3 and produce IFN-γ^44^. Similarly, Tregs expressing STAT3, RORγt, and CCR6 and producing IL-17 accumulate at sites of Th17-mediated inflammation, and the development of Th17 requires TGF-β^45^. We demonstrate that Nrp-1 and TGF-β can mutually affect Treg protein expression levels; however, IL-10 and Nrp-1 appear to result in enhanced augmentation of Th1 and Th17 immunity. TGF-β signaling seems to be less of a requirement for the suppression of Th1 and Th17 cell-mediated inflammation and its facilitation of tumor growth through increasing tumor-derived Nrp-1^+^ Treg cells. Above all, TGF-β plays a secondary role in TH immunity and the formation Treg cells compared with IL-10. Furthermore, we showed that, in contrast to IL-10, the absence of Nrp-1 had a marginal effect on TGF-β1; thus, blocking Nrp-1 will be much more efficient than blocking IL-10 to promote TH17 immunity. There appear to be important tissue- or inflammation-specific homeostatic cues that control Treg subpopulations. Treg expression was found to be important for the “competitive fitness” of the subpopulations by enabling their proliferation in Th1 and Th17-driven inflammatory conditions. Foxp3 is essential for Treg development and its suppressive activity partially through the maintenance of high levels of CD25 and CTLA4 expression. Treg development and survival are dependent on a number of key factors and signals, including IL-2, TGF-β, and co-stimulatory molecules such as CD28^46^.

Our studies demonstrated the importance of IL-10 and Nrp-1 for immunological processes in tumorigenesis, including Treg plasticity, stability and immunosuppressive functions. Tumor growth facilitates the induction or recruitment of CD4^+^ regulatory T cells that secrete IL-10 and TGF-β and suppress effector CD8 T cell responses^47^. IFN-r switches the balance of IL-10 STAT activation from STAT3 to STAT1 and acts as an important factor that impairs IL-10 immunosuppressive and anti-inflammatory activity^48^. STAT5 and Foxp3 are the lineage-specific STAT regulator and transcription factor for Treg cells, respectively. The important signature cytokine for Treg cells is TGF-β. Naive murine CD4^+^ T cells must be activated to become TH17 cells through the T cell receptor in the presence of TGF-β and IL-6. The lineage-specific STAT transcription factor and regulator for TH17 cells are RORrt and STAT3, respectively^49^. *In vivo*, IL-17A-producing CD4^+^ T cells express interleukin-10 receptor a (IL-10Ra) and are controlled by Foxp3^−^ and Foxp3^+^ regulatory CD4^+^ T cells in an interleukin-10-dependent manner^50^. Therefore, IL-10 plays an important role in the TH17/Treg balance. Two major classes of Tregs have been identified to date: CD4 and CD8 Tregs. CD4 Tregs consist of two types: Treg cells that either arise in the thymus and are called natural Treg (nTreg) cells, or Tregs generated in the periphery through the induction of Foxp3 that are called inducible Treg (iTreg) cells^51^. The infiltration of Foxp3^+^ regulatory T (Treg) cells is considered to be a critical step during tumor development and progression. The requirement of STAT3 for the expression of IL-10 has been demonstrated using several STAT knockdown mice. Treg is one source of IL10^19^. Transforming growth factor-β (TGF-β) induces Nrp-1 expression on Treg cells; however, this process can be prevented *in vitro* by the proinflammatory cytokine interleukin-6^9^. Interleukin-10 signaling in regulatory T cells is necessary for limiting Th17 cell-mediated inflammation^52^.

Finally, the prognostic and therapeutic potential of Tregs may depend not only on numbers or functions but also on their relative abundance^46^. The maintenance of immune homeostasis and the prevention of autoimmunity may not require Nrp-1 signaling^17^. Thus, NRPs fulfill several criteria for therapeutic targets for innovative anti-tumor therapies^12^,^53^. Possible combination blockades of IL10 and Nrp-1 via bispecific antibodies or soluble antagonists may one day be a viable therapeutic strategy to limit tumor-induced tolerance without evoking autoimmunity.

## Methods

### Mice

Six- to eight-week-old C57BL/6 IL10 KO mice were purchased from The Jackson Laboratory and bred in-house (Il10<tm1Cgn>Interleukin-10 Gene Targeted Mutation JAX Mice Database: 002251 B6.129P2-Il10 < tm1Cgn>/J). Wild type C57BL/6 mice were purchased from Vital River, Peking, China. Both of these mice were bred in-house under SPF conditions. The animal experiments were performed according to the guidelines of the Animal Care and Use Committee of Sichuan University (Chengdu, Sichuan, China) and approved by the Animal Care and Use Committee of Sichuan University (Chengdu, Sichuan, China).

### Cell culture and tumor induction of B16-F10 melanoma or E.G7-OVA cell lines

The murine melanoma cell lines B16-F10 and E.G7-OVA (an EL4 cell line transfected by electroporation with OVA cDNA to allow endogenous production of OVA with an H-2Kb-restricted CTL epitope) were obtained from the American Type Culture Collection (ATCC). B16/F10 melanoma cells were grown in DMEM Complete Growth Medium. E.G7-OVA cells were grown and maintained in RPMI 1640 medium supplemented with 10% fetal bovine serum (Gibco) and 400 μg/mL of G418. Both the B16-F10 and E.G7-OVA cell lines were incubated at 37 °C and 5% CO_2_. Mice were injected i.v. with 5 × 10^5^ B16/F10 cells. Two weeks post-inoculation, the mice were euthanized for enumeration of metastatic lung foci. All lung foci were evaluated under a tissue microscope (Leica MZFLIII). Additionally, a total of 1 × 10^6^ B16/F10 cells or 3 × 10^6^ E.G7-OVA cells were inoculated s.c. into the left flanks of IL-10^−/−^ and WT C57BL/6 mice. Tumor sizes were measured every 3 days using calipers fitted with a Vernier scale. At day +15, the mice were euthanized, and the tumors, spleens and TDLNs were harvested for flow cytometry and other experiments.

### Antibody neutralization *in vivo*

An antigen affinity-purified polyclonal IgG goat anti-mouse Nrp-1 antibody (AF566) and its isotype control (polyclonal goat IgG) (R&D Systems AB-108-C) were injected i.v. (1.8 mg/kg/mouse) into B16/F10 tumor-bearing mice^18^. Similar groups of mice were treated i.v. with either a rabbit anti-mouse TGF-β pan-specific polyclonal rabbit antibody (3 mg/kg; R&D Systems AB-100-NA) or an isotype-specific polyclonal rabbit IgG (3 mg/kg; R&D Systems AB-105-C)^54^. All antibody preparations were reconstituted in sterile PBS and then administered on days +9 and +12 post-B16/F10 implantation. Tumor sizes were monitored every three days (N = 3–6 mice per treatment group depending on the individual experiments presented in the results). The tumor, spleen and TDLN were harvested on day +15 to conduct the *in vitro* experiments outlined in the results.

### Single cell preparation for staining

The spleen, tumor or tumor-draining lymph node (TDLN) cell isolation procedure was based on our previously described procedure^55^. Tissues were dissected from freshly killed mice and trimmed of fat and connective tissue. Small cuts into the capsules were made with a pair of fine scissors, and the fragments were incubated in 1 mg/ml collagenase III (Gibco-Invitrogen) in RPMI 1640 medium at 37 °C for 15 min with gentle agitation using a Pasteur pipette every 5 min. Cells recovered by centrifugation were resuspended in FACS staining buffer (BD Pharmingen) and then passed through a 100-μm mesh strainer before counting and staining.

### Flow cytometry

The spleen, tumor or tumor-draining lymph node (TDLN) cell isolation procedure was based on our previously described protocol^55^. Tissues were cut into fragments and then incubated in 1 mg/ml collagenase III (Gibco-Invitrogen) in RPMI 1640 at 37 °C for 15 min. Flow cytometry staining was performed using fluorescence-conjugated anti–mouse antibodies targeting CD4, CD8a, CD69, CD25, IFN-γ, IL17A, CD11b and CD11c (BD Pharmingen). Cells were also incubated with goat anti-Nrp-1 (R&D Systems) and rat anti-TGF-β1 (Biolegend) followed by an Alex Fluor 647-conjugated anti-goat antibody (BD Pharmingen) or Alex Fluor 488-conjugated anti-rat antibody (BD Pharmingen). Intracellular staining was performed using a Foxp3 staining kit and fixation/permeabilization buffer (eBioscience). Samples were obtained using a FACSCalibur equipped with Cell Quest software (BD Biosciences). The cells were gated for lymphocytes based on the forward and side scatter profiles. All data were analyzed using Tree Star FlowJo software.

### Immunofluorescence analysis

Harvested tumor tissues were frozen in OCT and fixed with paraformaldehyde. Permeabilized tissues were stained with rabbit anti-mouse IL-10 (Abcam), followed by incubation with goat anti-rabbit IgG conjugated to Alexa Fluor 488 (BD Pharmingen). Nuclei were stained with 4′,6-diamidino-2-phenylindole (DAPI; Molecular Probes/Invitrogen). Fluorescent images were acquired using a fluorescence microscope (Leica).

### Immunohistochemical analysis of tumor tissue

After 15 days, the mice were euthanized, and the melanoma tumors were removed and fixed with 4% polyformaldehyde. Immunohistochemical (IHC) reactions were performed using the streptavidin peroxidase method. For the negative control, the slide was incubated in PBS. Primary antibodies against CD31 (ab28364), Ki-67 (ab15580), CD8a (ab4055), and Neuropilin 1 (ab81321) were applied according to the staining kit protocol. Biotinylated goat anti-rabbit and anti-rat antibodies (BOSTER SA1022 or SA1025) were used as the secondary antibodies. Images were recorded using a Leica DM 4000B photomicroscope. Image-Pro Plus 6.0 software (Media Cybernetics) was used to analyze the mean integrated optical density (mean IOD) of the target protein expression in tumor sections according to the following formula: mean IOD = IOD/area of the tumor section. Areas of necrosis and staining artifacts were excluded by AOI tools. Samples were stained from the tumor tissues of two to three mice per group and 3 to 4 slides by manual immunohistochemical (IHC) staining. Three images of each slide were assessed.

### Luminex immunoassay

Tumor tissues were harvested from tumor-bearing mice on day +15. The tumor-derived cytokines IL-6, VEGF (MCYTOMAG-70K), and multi-species TGF-β1 (TGF-β-64K-01) were measured using a mouse multiplexed, bead-based, immunoassay (MilliplexMapKit, Millipore Corporation). Tumor tissues were quantified in 25 μl of adipose tissue lysate (50 μg of protein in PBS containing 1% NP-40)^56^. Measurements were performed using the Luminex 100 System according to the manufacturer’s instructions (Bioplex System; Bio-Rad). The data were calculated using a log regression curve and a standard curve with respective recombinant proteins diluted in lysis buffer for tissue samples. Samples were assayed in duplicate to detect total protein (pg/ml). Total protein concentrations were determined using a BCA protein assay kit (ThermoScientific).

### Statistical analysis

All experiments were evaluated using one-way ANOVA for the determination of statistical significance with p < 0.05 between experimental groups. Some cases were evaluated with Student’s t-test. The results are expressed as the mean ± SD or mean ± SEM, where p < 0.05 was considered statistically significant. GraphPad Prism and SigmaPlot 12.5 software were used.

## Additional Information

**How to cite this article**: Wang, S. *et al*. Interleukin-10 deficiency impairs regulatory T cell derived neuropilin-1 functions and promotes Th1 and Th17 immunity. *Sci. Rep*. **6**, 24249; doi: 10.1038/srep24249 (2016).

## Supplementary Material

Supplementary Information
