# Supplementary material for: Interleukin-10 deficiency impairs regulatory T cell-derived neuropilin-1 functions and promotes Th1 and Th17 immunity
Source: Sci Rep. 2016 Apr 14;6:24249. doi: 10.1038/srep24249 (PMC4831052; doi:10.1038/srep24249)
Supplement: Supplementary Information [file srep24249-s1.doc]

**Interleukin-10 deficiency impairs regulatory T cell-derived neuropilin-1 functions and promotes Th1 and Th17 immunity**

Shimin Wang^1^, Xiang Gao^1,2*^, Guobo Shen^1^, Wei Wang^1^, Jingyu Li^1^, Jingyi Zhao^1^, Yu-Quan Wei^1*^, and Carl K. Edwards^1*^

^1^Department of Neurosurgery, State Key Laboratory of Biotherapy / Collaborative Innovation Center for Biotherapy, and West China Hospital, West China Medical School, Sichuan University, Chengdu, 610041, PR China

^2^Institute of Neurosurgery, West China Hospital, West China Medical School, Sichuan University, Chengdu, 610041, PR China

^*^**Corresponding authors:** Xiang Gao, Carl K. Edwards or Yu-quan Wei.

Email: ([**xiangxianggao2008@163.com**](mailto:xiangxianggao2008@163.com); Phone: ^+^86-28-85422136, Fax: ^+^86-28-85502796), ([**drcarledwards@gmail.com**](mailto:drcarledwards@gmail.com); Phone:^+^011-86-13551226910; Fax: ^+^011-86-28-85164)or ([**yuquanwei@scu.edu.cn**](mailto:yuquanwei@scu.edu.cn)**;** Phone: ^+^86-28-85502796, Fax: ^+^86-28-85502796)

**Competing financial interests:**The authors declare no competing financial interests.

**
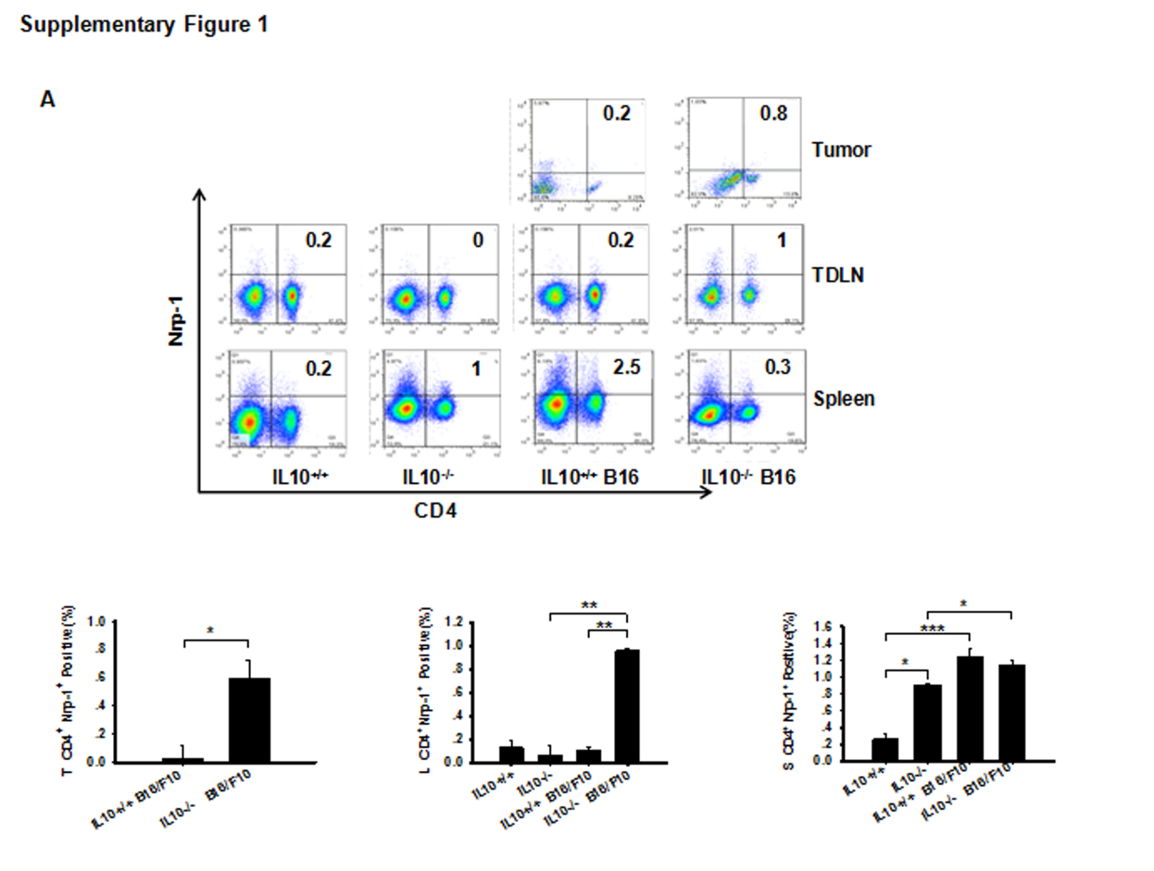
**

**Supplementary Figure 1:**

**IL10 deficiency affects CD4^+^ T cells producing Nrp-1 protein during melanoma tumor growth**

(A) Expression of CD4^+^Nrp-1^+^ cells in the tumor, TDLN and spleens of IL10^-/-^ B16/F10

versus WT B16/F10 mice by flow cytometry.

Tumor: Evaluated using student’s t-test for the determination of statistical significance with * P<0.05, between experimental groups. Data are expressed as mean values ± SEM from n=3-4 mice.

Spleen and TDLN: Evaluated using one way ANOVA test, *P<0.05, **P<0.005, ***P<0.001,± SEM from n=3-4 mice.


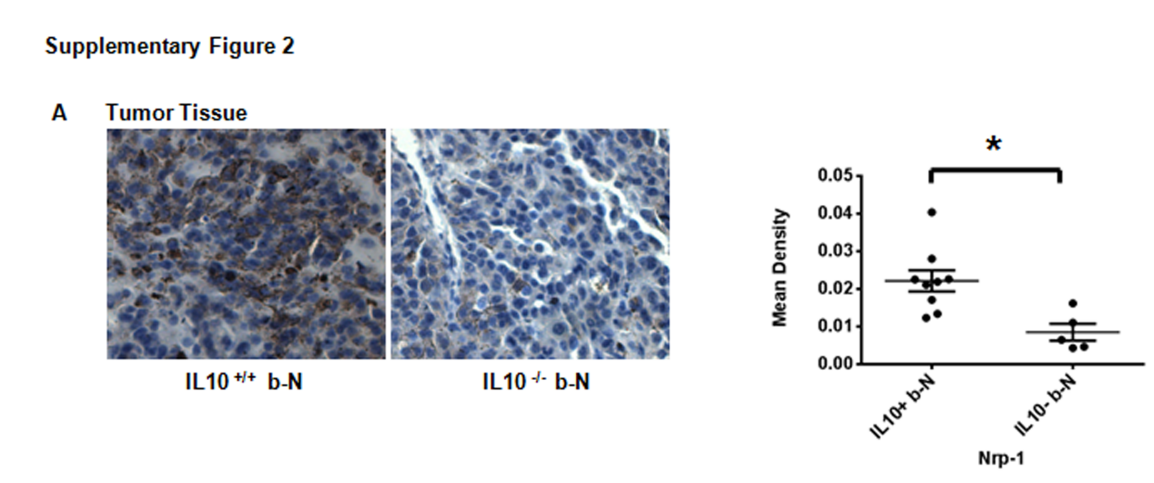


**Supplementary Figure 2:**

**Nrp-1 neutralization antibody efficiency worked in melanoma tumor model and increased CD8a expression to resist tumor progression**

1. Blocked Nrp-1 through i.v injection in the mice down-regulated Nrp-1 protein expression in tumor tissue. Representative photomicrographs of B16/F10 tumors harvested on day15.

Evaluated using student’s t-test for the determination of statistical significance with * P<0.05 between experimental groups. Data are expressed as mean values ± SEM from n=3-4 mice.

Panel A representative images of immunohistochemistry (left panel). Microscope with a 20X objective. Right panel, statistical results of the mean IOD for tumor cell proliferation analyzed by Image-Pro Plus software. Nonlinear regressions were carried out using GraphPad Prism (San Diego, CA).


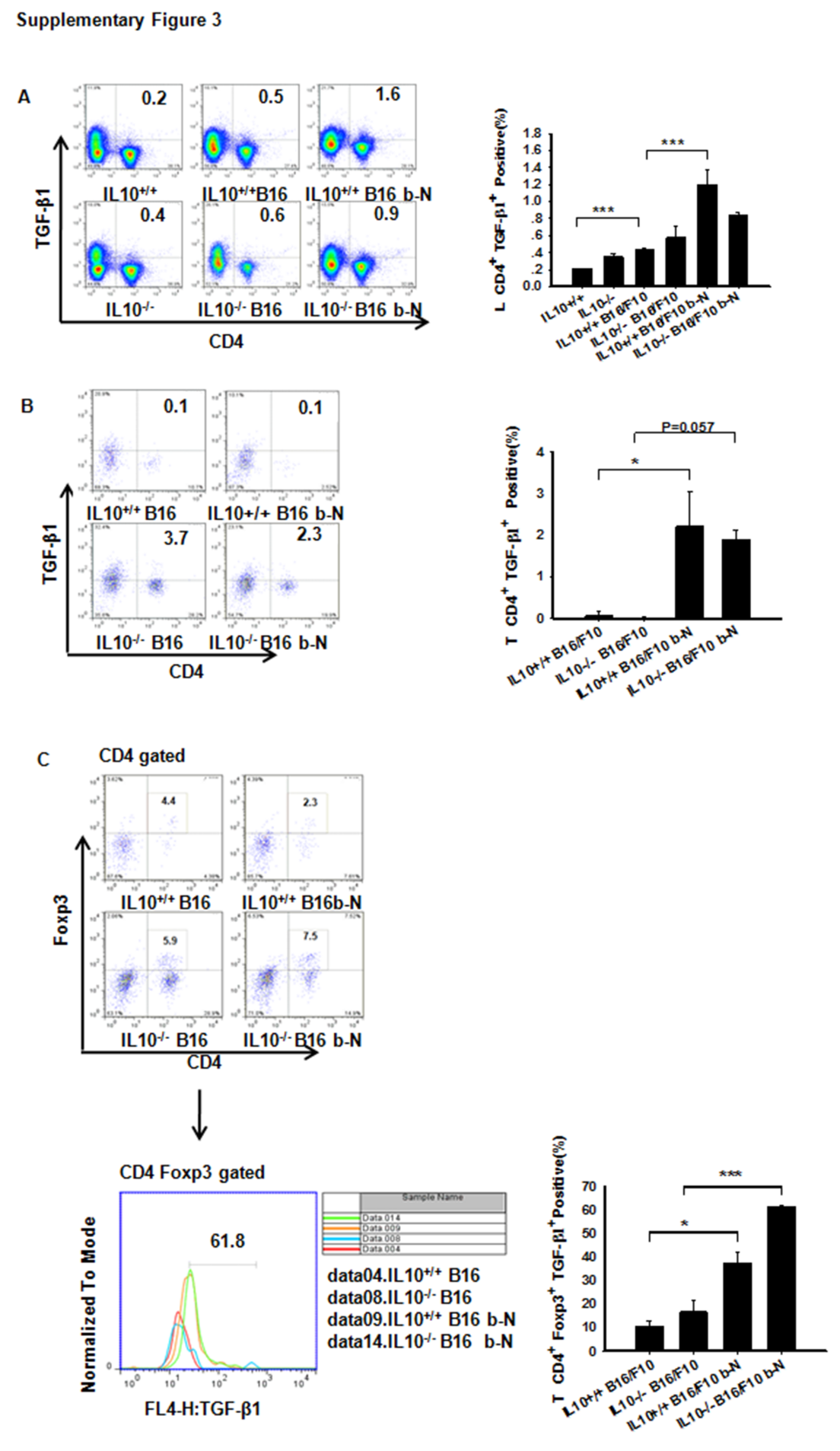


**Supplementary Figure 3:**

**Nrp-1 expression is more closely related to the number of Th3 and TGF-β1-producing Treg cells than IL-10 levels**

(A and B) Increased numbers of TDLN and tumor secreting CD4^+^TGF-β1^+^ T (Th3) cells in WT B16/F10 implanted mice treated with anti-Nrp-1 (d+15) by flow cytometry.

(C) Anti-Nrp-1 treatment augments tumor derived TGF-β1-producing Treg populations in WT and IL-10^-/-^B16/F10 mice compared with its control.

Panel A, B ,C and D: Evaluated using one way ANOVA test, *P<0.05 ,***P<0.001, *P<0.05, ± SEM from n=3-4 mice.


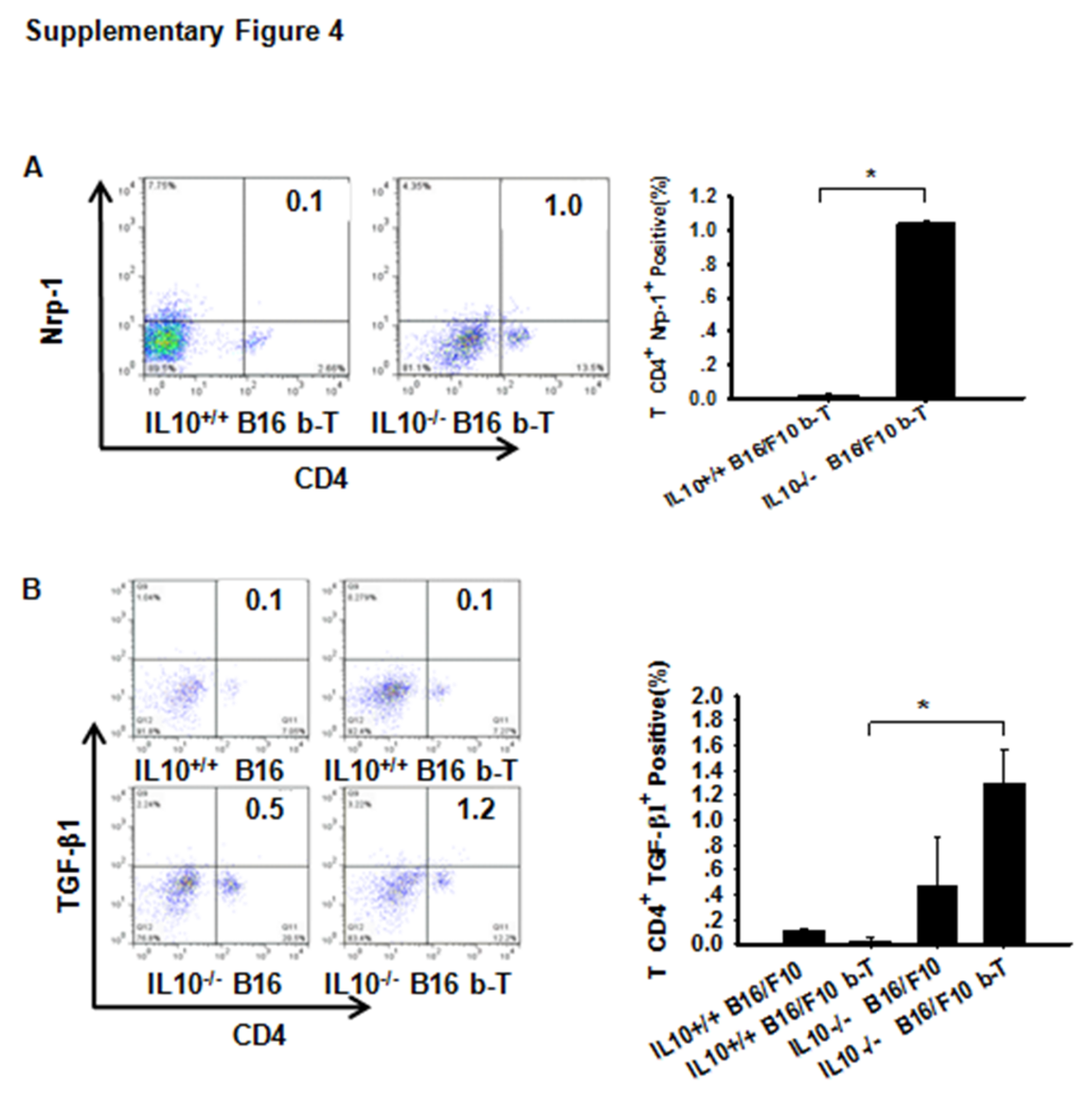


**Supplementary Figure 4:**

**Effect of TGF-β on CD4^+^ T cell population producing Nrp-1 protein and Th3 cells**

(A) Anti-TGF-β augments CD4^+^Nrp-1^+^ T cells in IL10^-/-^ tumor-bearing which measured by flow cytometry. Evaluated using student’s t-test for the determination of statistical significance with * P<0.05 between experimental groups. Data are expressed as mean values ± SEM from n=3-4 mice.

(B) Increased numbers of tumor secreting CD4^+^TGF-β1^+^（Th3）T cells in IL10-/- B16/F10 versus WT implanted mice, both treated with anti-TGF-β.

Evaluated using one way ANOVA test, *P<0.05 ,***P<0.001,± SEM from n=3-4 mice.


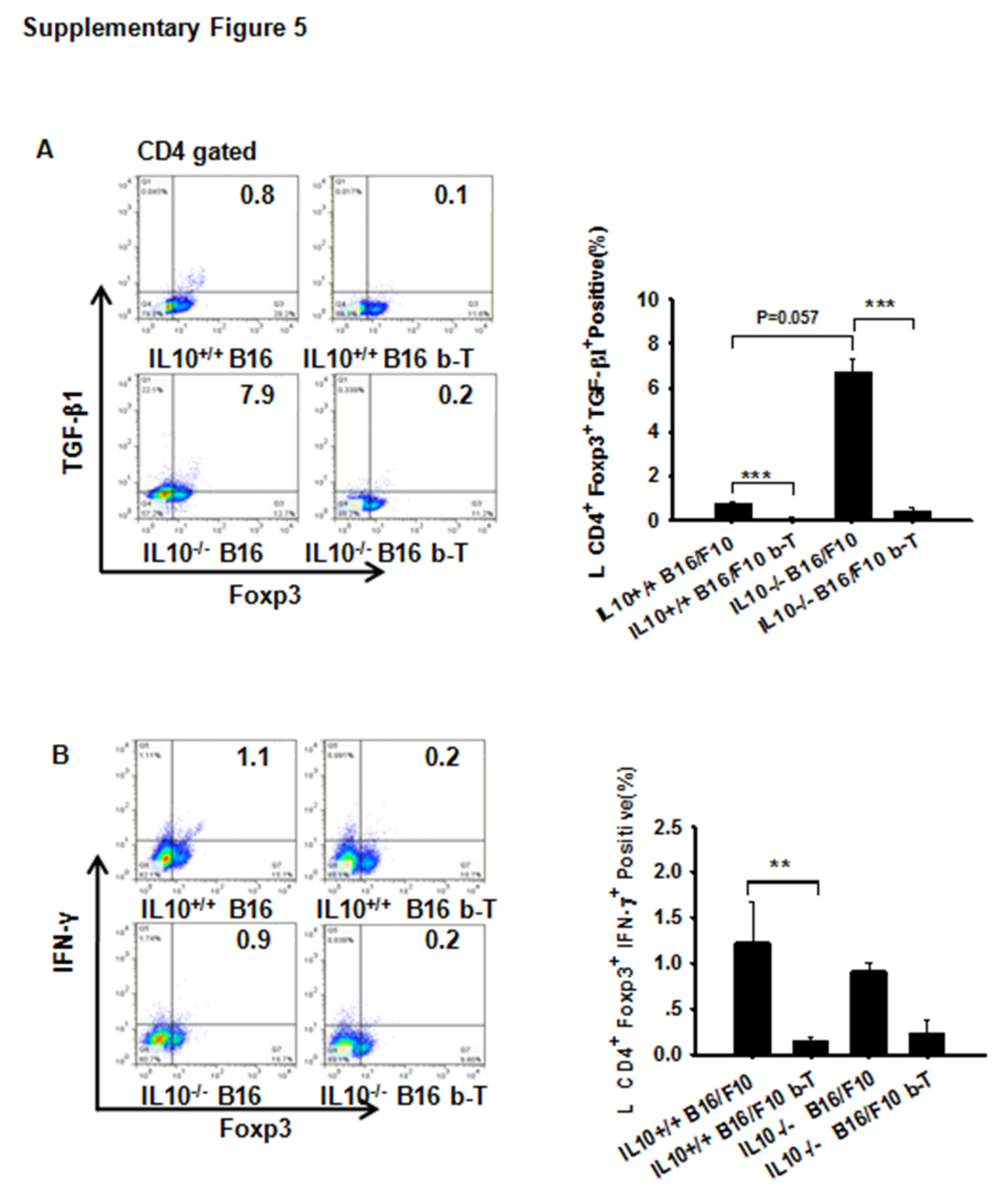


**Supplementary Figure 5:**

**Blocked TGF**-β1 **decreases Treg cells producing TGF-**β and I**FN-γin TDLN of melanoma tumor.**

(A and B) Anti-TGF-β treatment reduces the number of TDLN TGF-β1^+^ or IFN-γ^+^ CD4^+^Foxp3^+^cell populations in IL10^-/-^ B16/F10 mice and WT mice (day +15).

Evaluated using one way ANOVA test, *P<0.05 ,***P<0.001,± SEM from n=3-4 mice.
